# Visceral Leishmaniasis Caused by *Leishmania donovani* Zymodeme MON-37, Western Ghats, India 

**DOI:** 10.3201/eid2608.200557

**Published:** 2020-08

**Authors:** Prasanta Saini, N. Pradeep Kumar, P.M. Ajithlal, Aswathy Joji, K.R. Rajesh, K.J. Reena, Ashwani Kumar

**Affiliations:** Indian Council of Medical Research–Vector Control Research Centre (Field Station), Kottayam, India (P. Saini, N.P. Kumar, P.M. Ajithlal, A. Joji);; Government Medical College, Thrissur, India (K.R. Rajesh); District Medical Officer, Thrissur (K.J. Reena);; Indian Council of Medical Research–Vector Control Research Centre, Puducherry, India (A. Kumar); During 2015–2019, we recorded 10 patients with indigenous cases of visceral leishmaniasis caused by *Leishmania donovani* in Western Ghats, a region in India to which visceral leishmaniasis is not endemic. The parasite involved in 4 of these infections was of the MON-37 zymodeme strain, which normally causes cutaneous leishmaniasis in this region.

**Keywords:** Black fever, Dumdum fever, indigenous, kala-azar, lambda cyhalothrin, *Leishmania donovani*, leishmaniasis, liposomal amphotericin B, *Phlebotomus argentipes*, phlebotomine sand flies, visceral leishmaniasis, zymodeme MON-37, parasites

Leishmaniasis is a neglected tropical disease, caused by *Leishmania* parasites and transmitted by phlebotomine sand flies, which manifests in 3 primary clinical forms: visceral (VL), also known as kala-azar; cutaneous (CL); and mucocutaneous (*1*). The lack of continuous active surveillance, indefinite array of symptoms, resemblance to other infections, and diverse clinical manifestations may lead to misdiagnosis of this disease, especially in areas to which it is not endemic (*2*). Despite the reduction in VL reported by the National Kala-azar Elimination Programme, emergence or resurgence is being recorded in different regions of India (*3*,*4*). During 2003, two indigenous cases of VL were reported from Kerala (*5*). We report the occurrence of 10 additional indigenous cases of VL from the foothills of the Western Ghats in Kerala during March 2015–October 2019 (Figure). Ethics clearance for this study was obtained from the Indian Council of Medical Research–Vector Control Research Centre (approval no. IHEC-0119/R/M).

**Figure:**
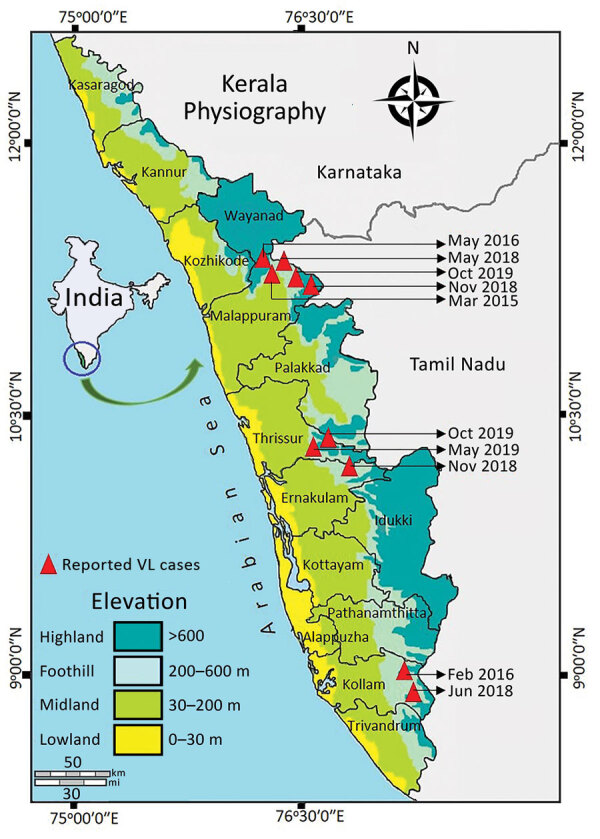
Spatial distribution and detailed timeline of VL cases in the foothills of Western Ghats, Kerala, India. VL, visceral leishmaniasis.

The patients exhibited clinical symptoms of VL, such as hepatosplenomegaly, fever, malaise, pancytopenia, anemia, emaciation, and anorexia. They tested negative for other microbial infections, such as HIV and tuberculosis. Histopathologic examination of bone marrow aspirates detected Leishman Donovan bodies within the macrophages. Results of serologic diagnosis with a Kalazar Detect rK39 rapid test kit (InBiOS, https://inbios.com) were also positive. We performed molecular diagnosis (*6*) on bone marrow and trephine biopsy samples from 4 of 10 patients. Among the other 6 patients, 3 were unwilling to provide samples; the other 3 patients died after diagnosis. 

PCR amplification of a kinetoplast DNA minicircle gene, restriction fragment-length polymorphism analysis of the 3ʹ untranslated region Hsp70 and DNA sequencing of Hsp70 gene amplicons (GenBank accession no. MT010559) characterized the parasites involved in the cases to be *Leishmania donovani*. Further genetic analysis of 6-phosphogluconate dehydrogenase gene sequences (A976G) indicated that the parasite belonged to the zymodeme MON-37 (GenBank accession no. MT010560). This strain is known to cause CL in the tribal belt of Western Ghats of Kerala (*6*) as well as in Sri Lanka (*7*). Thus, our investigations evinced that MON-37 zymodeme *L. donovani* is involved in both CL (*6*) and VL manifestations in the foothills of the Western Ghats, whereas MON-2 zymodeme of *L. donovani* caused VL in other VL-endemic zones across eastern India (*8*). Also, MON-37 has been characterized from an autochthonous VL case in Sri Lanka (*9*). These uncommon phenomena warrant further investigation. 

We treated the patients with an intravenous infusion of 1 dose of liposomal amphotericin B (10 mg/kg). Seven patients recovered completely; 1 patient, who was not responsive to the treatment and later died, was subsequently diagnosed with acute myeloid leukemia. Detailed treatment records were unavailable for the other 2 deceased patients. 

We monitored the recovered patients once every 3 months beginning with the commencement of treatment. We did not detect any relapse of symptoms or post–Kala-azar dermal leishmaniasis. Although rK39 antibodies have been documented to persist up to 5 years after treatment, immediate contacts of the VL patients tested negative for clinical symptoms of this disease and for rK39 antibodies by rK39 rapid diagnostic test. 

Entomologic investigations were carried out within a 0.5 km radius around the patients’ residences every 3 months for 1 year. *Phlebotomus argentipes* was the predominant sand fly species recorded. No natural infection with *Leishmania* parasites was detected in the sampled specimens; however, this result could be because of the limited number of cross-sectional entomologic surveys carried out and the long incubation period of *Leishmania* parasites. To control the vector population, indoor residual spraying with lambda cyhalothrin 10% wettable powder formulation was performed in and around houses, in all villages where cases had been reported. 

All of these patients lived in either tribal colonies or villages located in the foothills of Western Ghats, which encompasses one of the world’s major biodiversity hotspots. The region is characterized by its tropical climate, conserved forest ecosystem, low human activity, humid and shady microhabitats, and mud houses and is therefore highly conducive to sand fly breeding and proliferation. Epidemiologic surveys recorded that all of the patients lived in unplastered, humid, and poorly lit houses, which also serve as optimal resting sites for sand flies (*10*). The patients had histories of frequent visits to deep forest areas to collect forest products for their livelihoods. In addition, their reluctance to use protective clothing and insect repellents escalated their exposure to sand fly bites. Because of the difficulty of accessing these thickly forested regions, vector control activities using adulticidal insecticides have remained suspended in this region since the successful elimination of malaria during the 1960s.

None of the patients had a history of travel to other VL-endemic regions of the country or direct contact with persons from those regions, suggesting that these infections were indigenous. The occurrence of 10 VL cases in Kerala, a nonendemic state, during 2015–2019 suggests the need to adopt management strategies, including active surveillance, for leishmaniasis in the region. These actions would facilitate the successful implementation of guidelines from the ongoing National Kala-azar Elimination Programme in India. 
